# Fragrance in *Pandanus amaryllifolius* Roxb. Despite the Presence of a Betaine Aldehyde Dehydrogenase 2

**DOI:** 10.3390/ijms22136968

**Published:** 2021-06-28

**Authors:** Vacha Bhatt, Vitthal T. Barvkar, Agnelo Furtado, Robert J. Henry, Altafhusain Nadaf

**Affiliations:** 1 Department of Botany, Savitribai Phule Pune University, Pune 411007, India; vacha.biotech@gmail.com (V.B.); vbarvkar@gmail.com (V.T.B.); 2 Queensland Alliance for Agriculture and Food Innovation (QAAFI), The University of Queensland, St Lucia, QLD 4072, Australia; a.furtado@uq.edu.au (A.F.); robert.henry@uq.edu.au (R.J.H.)

**Keywords:** *P. amaryllifolius*, betaine aldehyde dehydrogenase 2, 2-acetyl-1-pyrroline, γ-aminobutyraldehyde

## Abstract

*Pandanus amaryllifolius* Roxb. accumulates the highest concentration of the major basmati aroma volatile 2-acetyl-1-pyrroline (2AP) in the plant kingdom. The expression of 2AP is correlated with the presence of a nonfunctional betaine aldehyde dehydrogenase 2(BADH2) in aromatic rice and other plant species. In the present study, a full-length *BADH2* sequence was reconstructed from the transcriptome data of leaf tissue from *P. amaryllifolius* seedlings. Based on this sequence, a 1509 bp coding sequence was defined that encoded a 54 kD PaBADH2 protein. This revealed the presence of a full-length BADH2 protein in *P. amaryllifolius*. Moreover, quantitative real-time PCR analysis, combined with BADH2 enzyme activity, confirmed the expression and functionality of the PaBADH2 protein. To understand the apparent structural variation, docking analysis was carried out in which protein showed a good affinity with both betaine aldehyde (BAD) and γ-aminobutyraldehyde (GAB-ald) as substrates. Overall, the analysis showed the presence of a functional *BADH2*, along with substantial 2AP synthesis (4.38 ppm). Therefore, we conclude that unlike all other plants studied to date, 2AP biosynthesis in *P. amaryllifolius* is not due to the inactivation of *BADH2*.

## 1. Introduction

Among the 750 Pandanus species, *Pandanus amaryllifolius* is the only species that produces the major basmati aroma compound 2-acetyl-1-pyrroline (2AP) in leaves. The species accumulates 2AP at the highest concentrations reported in the plant kingdom [1,2,3]. Leaves of *P. amaryllifolius* areroutinely used to flavor different food dishes in Southeast Asia [4]. In India, it was introduced in 1798, through the Botanical garden of Kolkata [5] and popularly cultivated along the Indian peninsular in kitchen gardens as a spice.

This species has been extensively studied in the laboratory of AN as follows. Bag-like structures called epidermal papillae were identified on the lower epidermis of the leaves of this species and act as a site of storage for 2AP [6]. Their pattern of development has been studied in the in vitro raised seedlings that showed that stomata act as the epicenter for its development [7]. A total of 21 new volatiles in this species have been identified [8]. Further, in vitro regeneration protocol has also been standardized. The populations of *P. amaryllifolius* grown under a wide range of environmental conditions across the Indian peninsular regions were collected and an elite population with the highest 2AP contents (12.2 ppm) was identified [9].These populations displayed remarkable genetic similarity.

We now report an investigation of the molecular basis of the extreme 2AP accumulation in this species. In aromatic rice and other plant species, 2AP synthesis is correlated with the presence of a nonfunctional *BADH2* gene [10]. A mutation in the *BADH2* gene introducing a premature stop codon causes loss of gene function and starts accumulating GAB-ald, a substrate of BADH2, and this GAB-ald spontaneously reacts with methylglyoxal to produce 2AP [11]. Fitzgerald et al. [12] reported that *BADH2* is playing a crucial role in maintaining the yield under salt stress and under exposure to stress conditions fragrant rice varieties with nonfunctional *BADH2* is more susceptible to yield loss. A detailed understanding of the enzymatic and molecular mechanism of *BADH2* in Pandanus can help to resolve the issue of stress susceptibility in fragrant rice. The BADH2 enzyme belongs to the aldehyde dehydrogenase (ALDH) super family. Members of this family possess substrate-binding, NAD^+^-binding, and oligomerization domains. The total loss of activity was observed associated with a mutation in Cys294 to alanine in the catalytic site of BADH2 [13,14,15]. Similarly, loss of activity was reported with a Glu260 to alanine mutation, and a reduced enzyme affinity was observed with an Asn162 to alanine mutation. Numerous studies have been carried out to identify the precursors of 2AP in various plant species. These studies reported that proline, ornithine, and glutamate act as the primary precursors of 2AP synthesis in *P. amaryllifolius* [16,17] and rice [18,19].

Although the economic importance of *P. amaryllifolius* is attributed to the aroma, limited information is available regarding the structure and specificity of BADH2 in this species. An earlier report [20] showed the presence of a truncated allelic form of the *BADH2* gene in in vitro plantlets of *P. amaryllifolius* (PND BADH2, gene ID: KY765936.1). Furthermore, in silico analysis suggested that insufficient affinity between the PaBADH2 enzyme and its substrate GAB-ald might be the major determinant of aroma development in *P. amaryllifolius*. Here, we obtained the full-length functional *PaBADH2* sequence from the transcriptome analysis and characterized its enzymatic activity, gene expression, protein sequence, active site, and interaction with a substrate.

## 2. Results

### 2.1. De Novo Transcriptome Assembly and Analysis

RNAseq data from the leaf of *P. amaryllifolius* was used to perform de novo assembly of the transcriptome. Raw data comprising 37,583,042 reads were obtained from the library. The SRA sequences were submitted to NCBI (BioProject ID: PRJNA692823). After removing the low-quality reads and all possible contamination, a total of 29,464,902 clean reads with Q ≥ 30 and 46% of GC content were obtained. Trinity-based de novo assembly generated contigs with N25 of 1663 bp, N50 of 1010 bp, and N75 of 555 bp. The N50 value of assembly is the sequence length of the shortest contig at 50% of the total transcriptome assembly length. It is a length-weighted median. N50 statistic defines assembly quality in terms of contiguity. Likewise, N25 and N75 are the minimum contig length to cover 25% and 75% of transcriptome assembly, respectively. A total of 34,672 transcript sequences were produced that had an average transcript size of 802 bp. A total of 27,734 transcripts had homologous genes in the NCBI nt database, while 6938 transcripts had no significant homology to any known genes. The assembly statistics are given in Supplementary Table S1.

### 2.2. Isolation of PaBADH2 Transcriptome Sequence and Its Comparison with Other BADH2 Sequences

**Figure 1:**
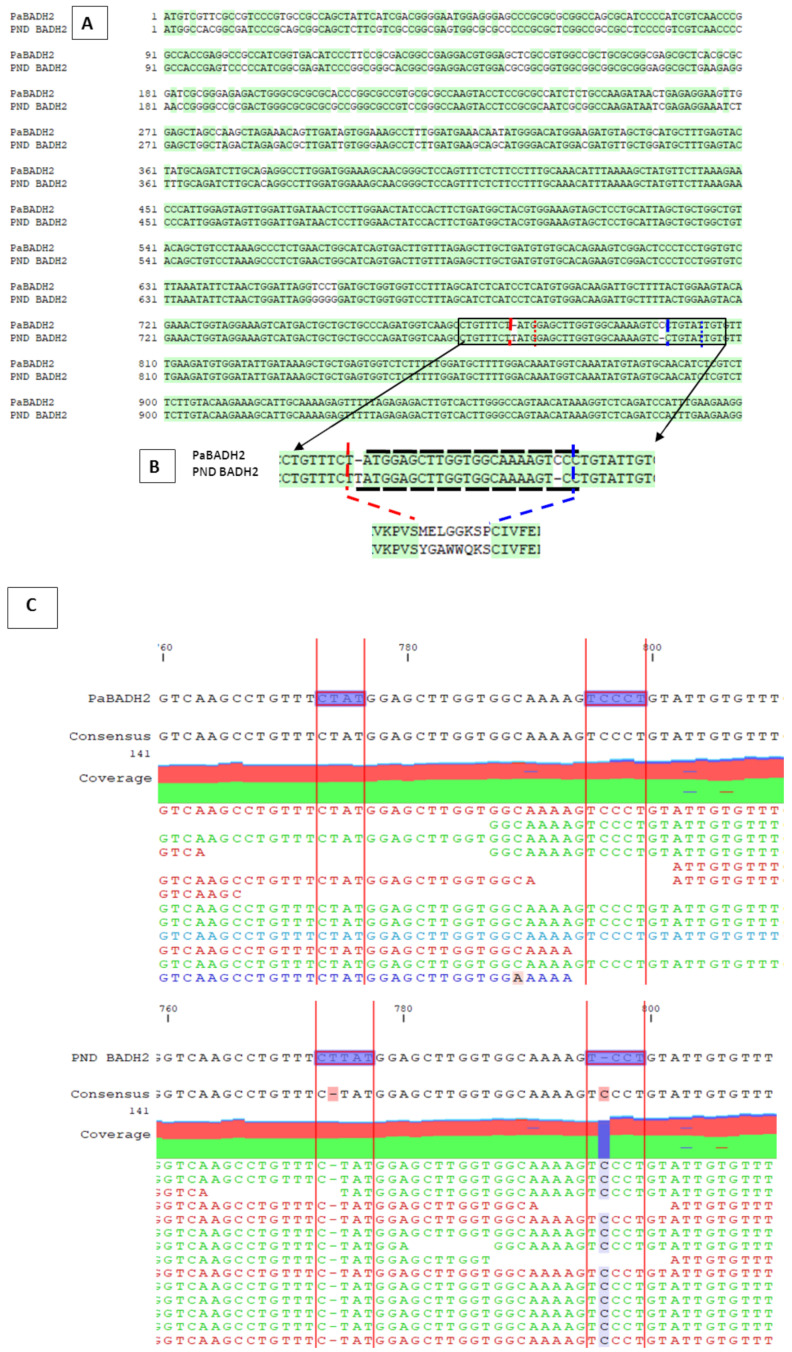
Mapping-based evidence for presence of the *PaBaDH2* gene in *P. amaryllifolius* genotype. *PaBADH2* and *PND BADH2*, *Betaine aldehyde dehydrogenase* gene of *Pandanus amaryllifolius* from the present study and Arora et al. (2017), respectively: (**A**,**B**) alignment of the *PaBADH2* and the *PND BADH2* genes showing several mismatches including the insertion of the T nucleotide and the deletion of the C nucleotide at each end of the 24 bp region indicated with red and blue broken vertical lines and the consequential change in the amino acid sequence; (**C**) mapping of whole-genome Illumina reads of *P. amaryllifolius* to the *PaBADH2* and the *PND BADH2* as reference sequences indicates the reads, showing no evidence of the T nucleotide insertion and the deletion of the C nucleotide at each end of the 24 bp sequence.

A full-length transcript sequence of *PaBADH2* was isolated from the assembled transcriptome using homology-based criteria and the transcript was found to be 1989 bp long. The sequence comprises a 5′ UTR from nucleotide 1 to 116 and a 3′ UTR from nucleotide 1629 to 1988. The gene sequence was compared with the previously reported *PND BADH2* and a nonscented rice *OsBADH2* sequence. There were several mismatches observed between *PaBADH2* and *PND BADH2* gene sequences. To confirm the reconstructed sequence, the raw sequencing reads were mapped onto the assembled BADH2 from *P. amaryllifolius* and this revealed that a total of 1762 reads mapped to the *PaBADH2* (1512 bp) sequence that supported the full-length sequence of an apparently functional transcript (Figure 1).

### 2.3. Comparative Amino Acid Sequence Analysis

The *PaBADH2* transcript included an open reading frame with 1509 nucleotides encoding a 503 amino acid full-length sequence, which would translate to a 54 kD functional protein. A peptide sequence (VSYGAWWQKS) and a cysteine residue (28th amino acid from the conserved peptide sequence), which was reported to be highly conserved in the proteins of aldehyde dehydrogenase, was observed in PaBADH2. Another characteristic conserved peptide, EGCRLGPVVS, reported in betaine dehydrogenases, was also present in PaBADH2. This finding suggests the presence of functional BADH2 protein in *P. amaryllifolius*. The *PaBADH2* gene contains a C-terminal SKL sequence, suggesting that the protein is targeted to the peroxisomes.

**Figure 2:**
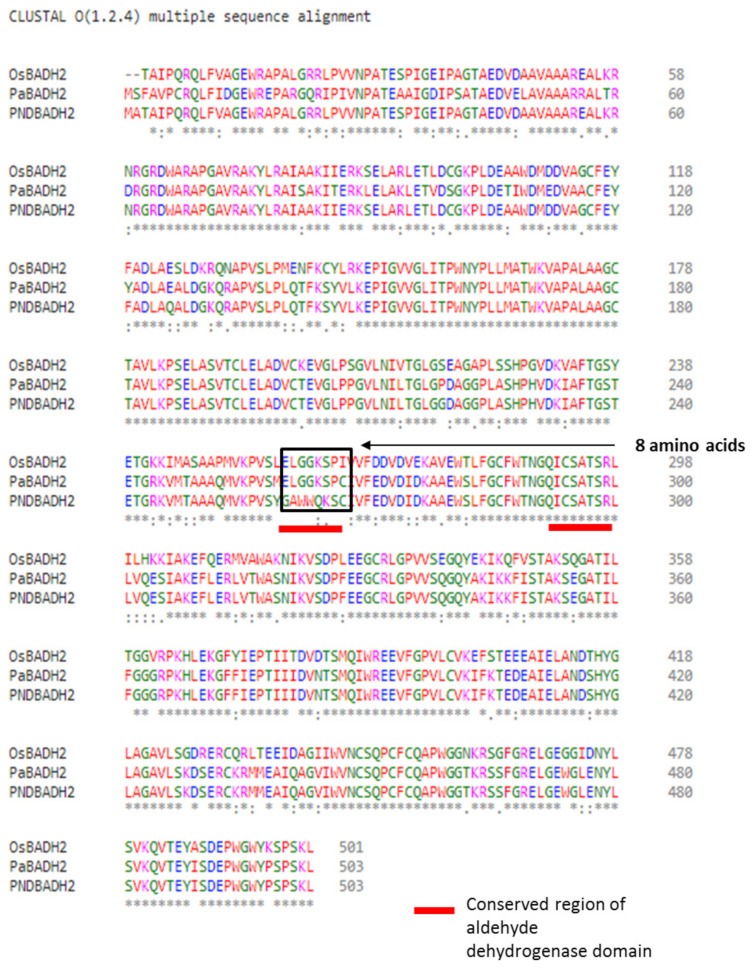
Multiple sequence alignment of BADH2 protein sequence from OsBADH2 (gene ID: 4345606), PND BADH2 (gene ID: KY765936.1), and PaBADH2. The marked region shows the continuous stretch of eight amino acid variations in PND BADH2, as compared with OsBADH2 and PaBADH2 sequences. The conserved region of the aldehyde dehydrogenase domain is represented with red lines.

The comparative sequence alignment of PaBADH2 and the previously reported sequence, PND BADH2 [20] exhibited 91.7% sequence similarity. The analysis also showed that most of the amino acid sequences were conserved between them except for a major consecutive 8 amino acid variation present at the middle of the PaBADH2 sequence. A critical amino acid of the catalytic domain of functional BADH2, Glu260, was present in PaBADH2, while it was mutated to glycine in PND BADH2. PaBADH2 showed 79.3% sequence identity with the rice sequence, OsBADH2 (Figure 2). Although there were some variations in the amino acid sequence of PaBADH2 relative to the OsBADH2 sequence, the formation of full-length protein was not hampered. The predicted amino acid sequence for PaBADH2 suggested the presence of a full-length functional protein because the differences in the sequences were not in the conserved amino acids that are important for enzyme activity.

### 2.4. Docking Analysis

**Figure 3:**
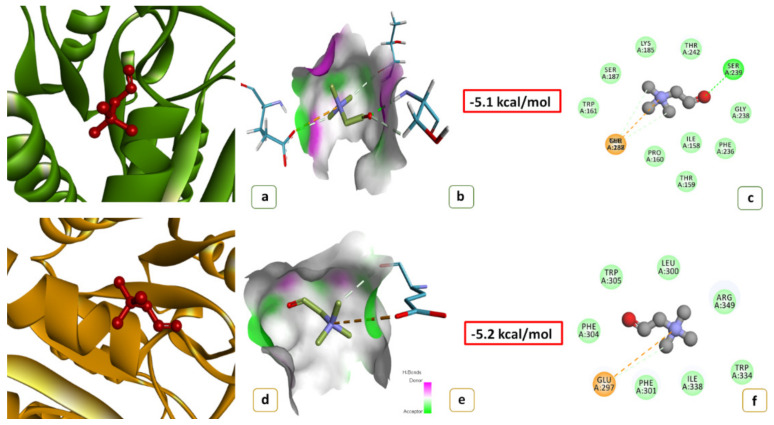
Docking analysis of PaBADH2 with betaine aldehyde (BAD): (**a**) docked 3D structure of PaBADH2-BAD complex; (**b**) showing H-bond donor (pink) and acceptor (green) surfaces in PaBADH2-BAD complex; (**c**) the 2D view of interacting residues of PaBADH2-BAD complex at ligand binding site that involves H-bond (green circle), Van der Waals bond (light green circle) and electrostatic interaction (brown circle); (**d**) docked 3D structure of OsBADH2-BAD complex; (**e**) showing H-bond donor (pink) and acceptor (green) surfaces in OsBADH2-BAD complex; (**f**) the 2D view of interacting residues of OsBADH2-BAD complex at ligand binding site that involves H-bonds (green circle), Van der Waals bonds (light green circle), and electrostatic interaction (brown circle).

**Figure 4:**
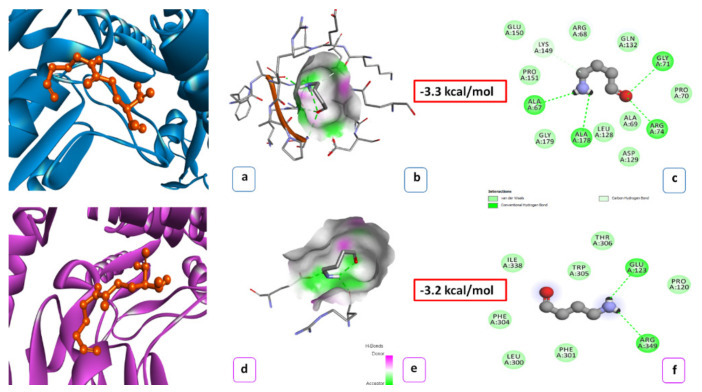
Docking analysis of PaBADH2 with 4-amino butaraldehyde (GAB-ald): (**a**) docked 3D structure of PaBADH2-GAB-ald complex; (**b**) showing H-bond donor (pink) and acceptor (green) surfaces in PaBADH2-GAB-ald complex; (**c**) the 2D view of interacting residues of PaBADH2- GAB-ald complex at ligand binding site that involves H-bond (green circle) and Van der Waals bond (light green circle); (**d**) docked 3D structure of OsBADH2 GAB-ald complex; (**e**) showing H-bond donor (pink) and acceptor (green) surfaces in OsBADH2GAB-ald complex; (**f**) the 2D view of interacting residues of OsBADH2-GAB-ald complex at ligand binding site that involves H-bonds (green circle) and Van der Waals bonds (light green circle).

To uncover possible functional differences between OsBADH2 and PaBADH2, protein docking analysis was carried out using BAD and GAB-ald as substrates (Figure 3 and Figure 4). The three-dimensional structure of the PaBADH2 protein was developed and enzyme–substrate interaction was studied using protein docking where BAD was used as the substrate for BADH2. With GAB-ald, the interaction energy for a monomer of PaBADH2 and OsBADH2 was −3.3 kcal/mole and −3.2 kcal/mole, respectively, whereas, in the case of BAD, it was −5.1 kcal/mole and −5.2 kcal/mole, respectively. The conserved amino acids of the NAD+ catalytic domains are almost identical in both proteins. The active site of PaBADH2 for substrate binding comprised of, Ile158, Thr159, Pro160, Lys185, Ser187, Glu188, Phe236, Gly238, Ser239, and Thr242. Likewise, the active site of OsBADH2 consists of Glu297, Leu300, Phe301, Phe304, Trp305, Trp334, Ile338, and Arg349. From this analysis, interactions were found in the complexes between hydrogen bonds, ionic bonds, C-H bonds, and Van der Waals interactions. The BAD interacted with the active site of PaBADH2 via two hydrogen bonds (2.1 A^0^), two C-H bonds (3.49 A^0^), one ionic interaction (4.64 A^0^), and six Van der Waals interactions (3.16 A^0^). In this reaction, the hydroxyl group containing amino acids Ser239 and Thr242 from the active site of the enzyme were associated in hydrogen bond formation with a ligand molecule. Moreover, the nucleophilic nature of the tertiary amine of the substrate allows the formation of an ionic bond with Glu188 present in the active site of the enzyme. Thr159 and Thr237 were present close to the substrate forming a strong C-H bond. Other than these, Phe236, Gly238, Lys185, Ser187, and Ile158 were involved in the Van der Waals interaction. In the same way, OsBADH2 also contained Glu297 that formed ionic interaction with the ligand, whereas other amino acids such as Leu300, Phe301, Phe304, Trp305, Trp334, Ile338, Arg349 were engaged in Van der Waals bond formation with the substrate. Overall, the docking analysis of both PaBADH2 and OsBADH2 suggested that there was not much difference in the interaction energies and the bond formation in the active site of both the enzymes; hence, it can be said that the PaBADH2 enzyme is likely to have similar activities.

### 2.5. BADH2 Enzyme Activity and Gene Expression Analysis

**Figure 5:**
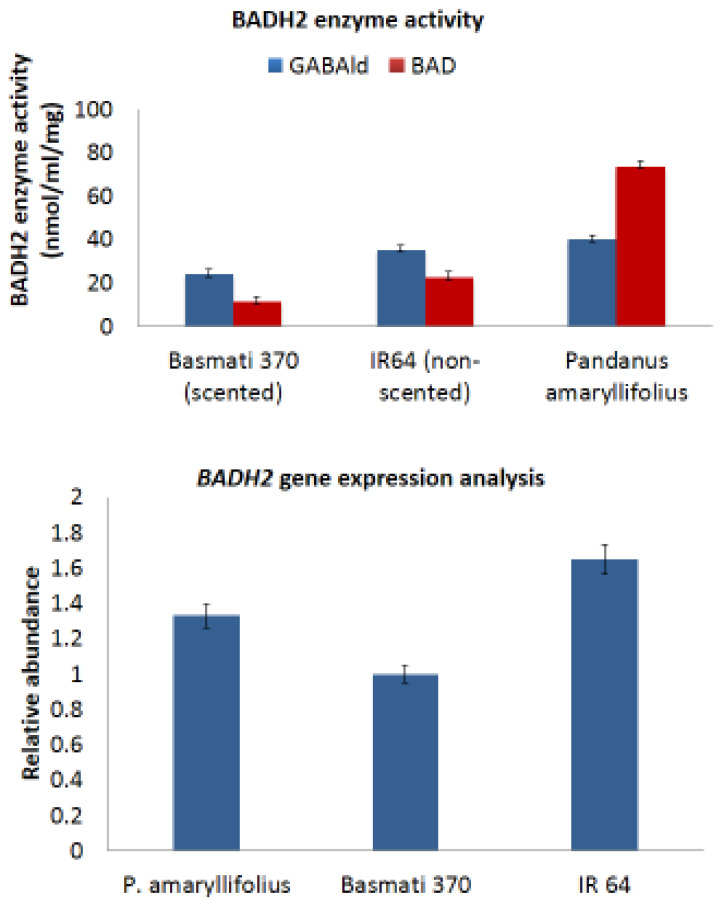
A comparative BADH2 enzyme activity using BAD and GAB-ald substrates and gene expression analysis in *P. amaryllifolius* and rice varieties. The *EF1* gene was taken as a reference gene for normalization and relative abundance (fold change) was calculated by the 2^−ΔΔCt^ method.

The BADH2 enzyme activity of *P. amaryllifolius*, Basmati 370 (scented rice), and IR 64 (nonscented rice) with GAB-ald as a substrate at pH 8.0 was 40.0 nM/min/g, 23.9 nM/min/g, and 35.1 nM/min/g, respectively (Figure 5). With BAD as a substrate, the same assay depicted a different trend. *P. amaryllifolius* showed 73.6 nM/min/g, Basmati 370 showed 11.4 nM/min/g, and IR 64 showed 22.5 nM/min/g enzyme activity. This confirms the presence of a functional BADH2 enzyme in *P. amaryllifolius*. Additionally, the study proves that *P. amaryllifolius* utilizes BAD substrate more efficiently than GAB-ald at pH 8 and vice versa for the rice varieties. The expression analysis of the *PaBADH2* gene (1.33 ±0.89 fold), *OsBADH2* in Basmati 370 (1.00 ± 0.081), and IR 64 (1.65 ± 0.98) showed that it was upregulated in leaf tissue (Figure 4). This result further highlights the presence of the functional *PaBADH2* gene in *P. amaryllifolius*.

### 2.6. Quantification of 2AP and Other Metabolites

**Figure 6:**
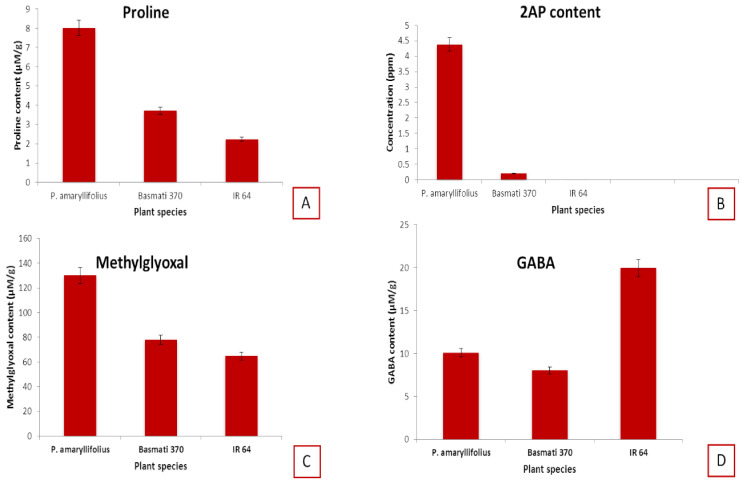
Comparative metabolite analysis in *P. amaryllifolius*, Basmati 370, and IR 64. The graph shows a comparative analysis of four key metabolites, namely, (**A**) proline, (**B**) 2AP, (**C**) methylglyoxal, and (**D**) GABA of 2AP biosynthetic pathway. Analysis was carried out in triplicates, and the standard deviation is represented with error bars. (Mean ± SE, *n* = 3).

The highest amount of 2AP was recorded in *P. amaryllifolius* (4.38 ppm), followed by Basmati 370 (0.20 ppm) and the absence of 2AP in IR 64 (Figure 6). The amount of 2AP in *P. amaryllifolius* was 20–22 times higher than that in the basmati rice varieties. Thus, *P. amaryllifolius* produces significantly higher concentrations of 2AP than fragrant rice. These results are in agreement with earlier reports [8]. Among the metabolites studied, free proline (8.01 μmoles/g) and methylglyoxal (130 ± 0.020 μM/g) contents were highest in *P. amaryllifolius*. Yadav et al. [21] reported 62.3 and 74.8 μM/g of methylglyoxal in rice varieties IR 64 and PB1. This indicates that *P. amaryllifolius* contains an appreciably higher amount of methylglyoxal, as compared to nonscented rice varieties. Moreover, the same trend was followed by GABA content (10.1 µg/g). The concentration of GABA in various plant parts commonly ranges from 0.03 to 5.5 µg/g [22]. Rashmi and Nadaf [23] reported 15.0 µmoles/g GABA in *P. odorifer* plants. The amount of GABA present in *P. amaryllifolius* was also considerably higher than in other plants.

### 2.7. Enzyme Activity of Δ1-Pyrroline-5-Carboxylate Synthetase (P5CS) and Expression Analysis

**Figure 7:**
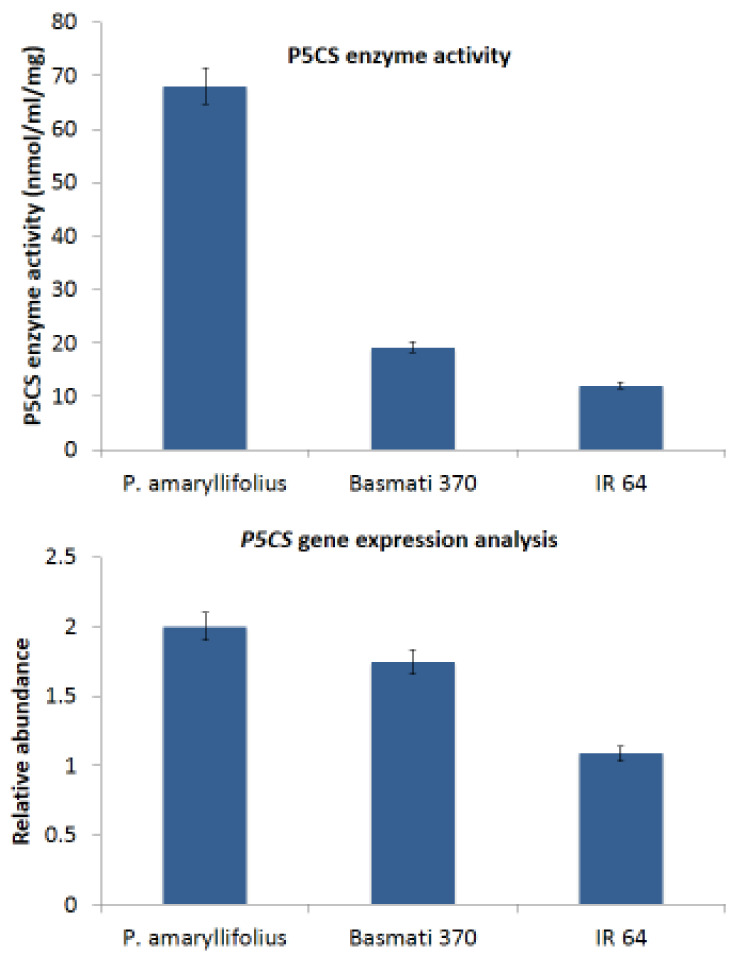
A comparative P5CS enzyme activity and gene expression analysis in *P. amaryllifolius* and rice varieties was carried out. The *EF1* gene was taken as a reference gene for normalization and relative abundance was calculated by the 2^−ΔΔCt^ method.

The P5CS enzyme activity and transcript levels were found to be higher in *P. amaryllifolius* (67.8 ± 2.8 nM/min/g and 2.00 ± 0.71 fold, respectively), which further correlates with the higher amount of proline and 2AP (Figure 7). P5CS enzyme is related to the enhancement of Δ1-pyrroline-5-carboxylic acid (P5C), thus resulting in the accumulation of higher 2AP. Kaikavoosi et al. [24] reported that overexpression of the *P5CS* gene enhanced the accumulation of proline, P5C, and 2AP in aromatic rice.

## 3. Discussion

### 3.1. De Novo Transcriptomics Analysis

De novo assembly and assessment of IIIuminaRNAseq data from *P. amaryllifolius* yielded a total of 27 million reads. Rashmi et al. [25] reported 28 million reads in *P. odorifer*, a species from the same genus. Both species have almost the same depth of sequencing. RNA seq data of *P. odorifer* showed an average transcript size of 951 bp and N50 of 1821 bp, whereas in *P. amaryllifolius*, the average transcript size was 802 bp and N50 of 1010 bp. This indicates that the average transcript size of *P. odorifer* is slightly longer than that of *P. amaryllifolius*, while the N50 value of the contigs showed variation in size.

### 3.2. P. amaryllifolius Exhibits Functional BADH2 Gene

One of the aims of the present study was the isolation and sequence characterization of *PaBADH2* in *P. amaryllifolius*. The transcriptome analysis clearly revealed the expression of *PaBADH2* with a single open reading frame without any stop codons. Further, the sequence alignment with functional *OsBADH2* revealed over 79.3% sequence similarity. The homology between the conserved BADH2 domains indicated that this was a functional sequence. Arora et al. [20] reported *BADH2* transcripts from in vitro *P. amaryllifolius* plantlets. When both the sequences were aligned, the length of the *PaBADH2* sequence differed from the *PND BADH2* gene sequence. This may be due to the presence of 5′ UTR and 3′ UTR. The sequence derived by transcriptome analysis showed the presence of 5′ UTR from nucleotides 1 to 116 and 3′ UTR from nucleotides 1629 to 1988. Further, the alignment of the *PaBADH2* and the *PND BADH2* genes showed several mismatches with a prominent variation of 24 base pairs, as depicted in Figure 1. The several mismatches showed the insertion of a T nucleotide and the deletion of a C nucleotide at each end of the 24 bp region. The mapping of transcriptome reads to *PaBADH2* and the *PND BADH2* as reference sequences indicated that the reads show no evidence of the T nucleotide insertion and the deletion of the C nucleotide at each end of the 24 bp sequence. This analysis indicated the distinctness of this sequence from *PND BADH2*.

### 3.3. Comparative Amino Acid Sequence Analysis

Our data report the presence of a functional BADH2 protein in *P. amaryllifolius*. The comparative amino acid sequence alignment between PaBADH2 and OsBADH2 showed that there is no variation in conserved amino acids. Similar to OsBADH2, all three key amino acids are present in PaBADH2. These are not directly involved in the protein–ligand interaction but provide a suitable framework that mediates the catalytic activity. As reported by Arora et al. [20], *PND BADH2* does not produce a functional protein. The sequence alignment showed that in the PND BADH2 amino acid sequence, a crucial amino acid Glu260 is replaced by Glycine [20]. The conversion of acidic amino acid into a hydrophobic amino acid may be the reason that made PND BADH2 unable to form a suitable cavity for ligand and therefore explain why PND BADH2 might be nonfunctional. In contrast, the key amino acids involved in the reaction are conserved in PaBADH2 making it functional. Therefore, it can be inferred that the presence of conserved amino acids helped in the formation of the required cavity for the substrate, and the binding residues of the enzyme located at the exposed region provided a favorable interaction with the substrate. Hence, the PaBADH2 protein present in *P. amaryllifolius* showed functionality as it exhibits affinity and binding capacity with the substrate.

### 3.4. Docking Analysis

The variations in amino acid sequence were further studied by structural characterization of PaBADH2 protein through docking analysis. A comparative PaBADH2 and OsBADH2 homology modeling showed that the alignment of both models resulted in a 0.115 RMSD value. That claims that the 3D structures of both the proteins are closely similar without any major differences. That shows changes in amino acid sequence do not alter the 3D structure of the protein. To verify that it does not hamper the interaction between protein and substrate, docking analyses were carried out. In BADH2 enzymes, the NAD+ binding domain is mainly responsible for the conversion of intermediate aldehydes to their respective carboxylic acids. The crystal structure of AMADH from *Spinacia oleracea, Pisum sativum* [26], and *Oryza sativa* [27] have been studied. The 3D structure and simulation studies of OsBADH2 enzyme carried out by Baicharoen, Vijayan, and Pongprayoon [27] inferred that Asn162, Glu260, and Cys294 are prominent amino acids of the catalytic domains. The previous study in rice suggested this catalytic triad as crucial for the catalytic activity of ALDH. These three amino acids are conserved in many plant species. In the hemithioacetal enzyme formation, Cys294 and Glu260 are involved and Asn162 helps in the stabilization of an intermediate. In the protein–ligand interaction, the oligomerization domain shows little mobility, while during the interaction, the NAD+ domain shows maximum mobility. Asn162 and Glu260 form strong hydrogen bonds with nearby residues, resulting in a rigid conformation. The strong interaction network may help to shape a suitable cavity size and dimension that fits both substrate and cofactor. In contrast, Cys294 remained mobile inside the pocket. Numerous reports are available that show that mutation in any of the amino acids from this catalytic domain results in reduced enzyme-substrate affinity. The conversion of Asn162 to alanine resulted in a momentous reduction in enzyme affinity with the substrate, while a change of Glu260 to alanine led to a complete loss of enzyme activity [15,28,29]. Mutation in Cys294 to alanine was reported to entirely abolish the activity of the enzyme [13,14]. Kamaraj and Purohit [30] executed a comparative rice BADH2 enzyme interaction study using rice mutant lines BADH2^N162A^, BADH2^E260A^, and BADH2^C294A^. The docking analysis revealed that the protein structure developed by mutant lines showed a narrower binding pocket than the wild-type BADH2 protein, and it also exhibited lower interaction energy when docked with GAB-ald. The minimum enzyme interaction was found in BADH2^N162A^, followed by BADH2^E260A^ and BADH2^C294A^. Therefore, it was concluded that these conserved amino acids play a major role in the establishment of appropriate binding cavity for better accommodation of the substrate. Any further changes in these conserved amino acids lead to change in the dynamics of the binding cavity, which, in turn, affects the enzyme–substrate interaction. Arora, Sultana, Kumar and Gangopadhyay [20] reported docking analysis in which they showed E260 as a conserved amino acid of substrate binding pocket; however, it is missing in their PND BADH2 sequence. The same amino acid is present in OsBADH2 and PaBADH2 sequences. Further, in the sequence, they reported glycine in place of glutamate (E260). This questions the authenticity of their sequence. In contrast, the docking analysis of our sequence confirms the functionality of PaBADH2.

The high PaBADH2 enzyme activity in vivo (46 nM/min/g) confirmed these findings. Srivong et al. [31] reported BADH2 enzyme activity in aromatic (Khao Dawk Mali 105) and nonaromatic rice (Sew-Mae- Pah-Tawng) at 26 nmol/min and 37 nmol/min, respectively. These results revealed higher BADH2 activity in P. amaryllifolius than in rice. GAB-ald and BAD are specific substrates of BADH2. Arakawa et al. [32] have also reported BADH2 activity in barley, wheat, and spinach as 0.17, 0.22, and 0.82 nM/min/mg of total protein, respectively. These data support the existence of a functional BADH2 in P. amaryllifolius. Further, the qPCR analysis also revealed the existence of the *BADH2* gene at the transcript level, which supports the functionality of the BADH2 protein.

### 3.5. Role of Precursors in the High Expression of 2AP in P. amaryllifolius

*P. amaryllifolius* is a potent producer of 2AP. The *P. amaryllifolius* leaf tissue showed the presence of 2AP. Basmati rice has been reported to accumulate up to 1 ppm of 2AP and in the highly scented rice variety Khao Dawk Mali-105 up to 3 ppm of 2AP [33]. Yoshihashi, Nguyen, and Kabaki [19] reported relatively higher 2AP in the aerial parts of the rice plants when compared to the milled grains. Moreover, 2AP has been reported to be synthesized throughout rice plants except in the roots [34]. Similar to the reports for aromatic rice, the reports for 2AP indicate that it is synthesized in aerial parts of *P. amaryllifolius*. The presence of 2AP prompted us to analyze the precursors related to the 2AP biosynthetic pathway. Proline has been identified as a precursor amino acid for the biosynthesis of 2AP and higher concentrations have been reported to contribute to enhanced 2AP levels. Several reports have demonstrated the role of proline in 2AP pathway. Using tracer experiments with ^15^N-proline, Huang et al. [34] demonstrated that the nitrogen of 2AP was derived from proline. Thimmaraju et al. [17] reported enhanced production of 2AP by feeding L-proline to the semi-differentiated callus culture of *P. amaryllifolius*. The maximum level of 22 mg/kg of 2AP was synthesized when rice calli were treated with 1 mmol/l of L-proline. Suprasanna et al. [18] demonstrated that proline addition enhanced the aroma in callus cultures of aromatic rice, suggesting that proline was related to aroma synthesis. Kaikavoosi et al. [24] overexpressed the *P5CS* gene in scented rice in order to achieve enhanced accumulation of proline, P5C, and 2AP. The leaf samples accumulated 8.07 ± 0.09 μmoles/g of free proline and a higher amount of P5CS enzyme activity 67.8 ± 2.83 with the presence of transcripts supporting the higher accumulation of 2AP in *P. amaryllifolius* leaves.

In addition to proline, methylglyoxal has also been identified as another precursor for the biosynthesis of 2AP. Wu et al. [35] reported higher (1.9 μmol/g) MG levels in aromatic than nonaromatic cultivars (1.2 μmol/g) of soybean. MG was identified as the carbon source for 2AP. Vegetable soybeans with higher levels of 2AP were reported to contain higher amounts of MG demonstrating a direct relation between MG and 2AP. They also demonstrated that an intermediate compound derived from proline reacts nonenzymatically with MG to produce 2AP.

In addition, the synthesis of GABA is regulated by *BADH2*. In aromatic rice varieties, since *BADH2* is nonfunctional, low GABA synthesis takes place. However, the amount of GABA present in *P. amaryllifolius* was considerably higher. The concentrations of GABA in various plant parts commonly range from 0.03 to 5.5 µg/g fresh weight [22]. This study demonstrated higher levels of proline and methylglyoxal that might be responsible for the high 2AP expression in *P. amaryllifolius*. BADH2 enzyme activity leads to an increased level of its substrate, GAB-ald, the immediate precursor of 2AP [36]. Utilization of GAB-ald by converting it into GABA blocks 2AP synthesis, whereas the enhancement of GAB-ald results in increased 2AP synthesis.

### 3.6. BADH2-Independent 2AP Synthesis in P. amaryllifolius?

**Figure 8:**
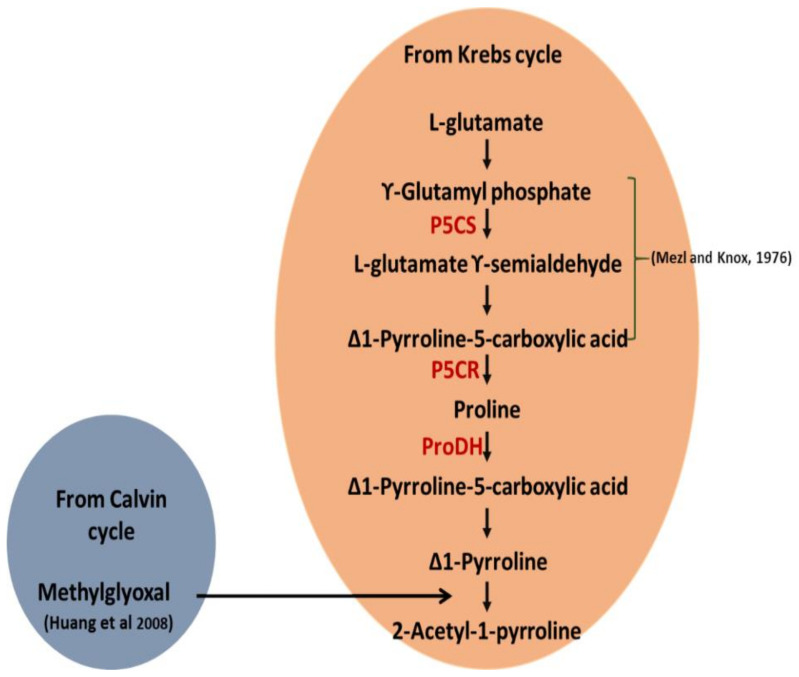
Proposed 2AP biosynthetic pathway in *P. amaryllifolius*, highlighting enzymes used in biosynthesis of 2AP in *P. amaryllifolius.P5CS*: Δ1-pyrroline-5-carboxylate synthetase; P5CR: Δ1-pyrroline-5-carboxylate reductase; ProDH: proline dehydrogenase.

Considering the functionality of *PaBADH2* in *P. amaryllifolius*,along with high 2AP synthesis, raises the question of how 2AP is synthesized in *P. amaryllifolius*. Interestingly, Huang et al. [34] reported that in aromatic rice, the upregulation of *P5CS* might be associated with the higher levels of delta-1-pyrroline-5-carboxylic acid, thus showing accumulation of a high amount of 2AP. The analysis of the expression level of *Δ^1^-pyrroline-5-carboxylic acid synthetase* and the enhanced concentration of its product among fragrant and nonfragrant varieties exhibited that 2AP is synthesized by the direct reaction of methylglyoxalwithdelta-1-pyrroline-5-carboxylate independent of BADH2 [37]. In another study, Huang et al. [34] studied an in vitro model system and demonstrated that methylglyoxal was one of the main precursors for 2AP synthesis. Methylglyoxal might react directly with ∆^1^ -pyrroline-5- carboxylic acid, derived from L-proline by purified recombinant B. subtilis ssp. natto PRODH, and lead to the formation of 2-acetyl-1-pyrroline. The overproduced ∆^1^-pyrroline-5-carboxylic acid may be detoxified by methylglyoxal and results in the biosynthesis of 2AP. In our analysis, we recorded comparatively higher levels of P5CS and methylglyoxal in *P. amaryllifolius*. Therefore, ultimately, this mechanism might be existing in *P. amaryllifolius*, which might be responsible for high 2AP synthesis independent of *PaBADH2* (Figure 8).

Moreover, fragrance is a trait of high value in rice. The Discovery of the recessive fragrance gene in rice Bradbury et al. [10] has facilitated the breeding of fragrant rice. The same gene has been linked with fragrance in sorghum and other plant species. The loss of function of the *BADH2* gene [10] that results in fragrance is associated with a loss of performance that may be due to stress susceptibility caused by the disruption of a pathway that is a key stress response mechanism in plants [12]. Pandanus leaves have the same fragrance but are much more resistant to stresses, such as salt [38], than fragrant rice varieties. Understanding the biochemical processes in Pandanus may support the breeding of higher-yielding stress-tolerant rice and other crops that are also fragrant.

## 4. Material and Methods

### 4.1. Plant Material

One-year-old seedlings of *P. amaryllifolius*,maintained at the Botanical garden of Department of Botany, Savitribai Phule Pune University, Pune, Maharashtra, were used for transcriptome analysis, estimation of metabolites, enzyme assay, and gene expression analysis. The seeds of fragrant (Basmati 370) and nonfragrant (IR64) rice cultivars were obtained from the Maval region, Maharashtra, and Indian agriculture research institute (IARI) New Delhi, India, respectively. For the estimation of metabolites, enzyme assay, and gene expression analysis, leaves of 45-day-old rice seedlings and one-year-old plantlets of *P. amaryllifolius* were used.

### 4.2. Transcriptome Analysis

For transcriptome analysis, total RNA was extracted from the leaf tissue of *P. amaryllifolius* using a Qiagen RNeasy Plant Mini Kit (Qiagen, Germany), following the manufacturer’s protocol, and DNase I treatment at 37 °C for 30 min was used to remove any potential genomic DNA contamination. The quality and quantity of isolated RNA were checked using a Nanodrop (ND-1000) spectrophotometer (Thermo Fisher Scientific, Waltham, MA, USA) and by analyzing the RNA samples on a 1% agarose gel. The quality of the RNA samples was further checked on a Bioanalyzer 2100 (Agilent technologies, Singapore). A sample with an RNA integrity number (RIN) of more than 7 was used for RNA sequencing analysis.

### 4.3. RNA Sequencing, De Novo Assembly, and Functional Annotation of Unigenes

Paired-end libraries of 150bp read length were sequenced using a HiSeq 2500 system (Illumina, San Diego, CA, USA) by SciGenom, Kochi, India. The raw sequence data generated were analyzed through the following steps. The raw data were filtered using an NGSQC Toolkit (v2.3.3 NIPGR, New Delhi, India) to remove adaptor sequences and reads with low quality (Phred) scores (Q ≥ 30). Only high-quality reads were further used for de novo transcriptome assembly using Trinity (v2.2) pipeline [39]. The resulting trinity assembly was made non-redundant using CD-HIT (v4.6.1). The assembled unigene sequences were annotated using BLASTx (E-value ≤ 1E^−5^) (http://www.ncbi.nlm.nih.gov/BLAST/) (accessed on 18 February 2019) against the National Center for Biotechnology Information (NCBI) database of non redundant protein (Nr) (http://www.ncbi.nlm.nih.gov) (accessed on 18 February 2019) [40].

### 4.4. Isolation of PaBADH2 Gene Using Transcriptome Sequence

A standalone blastable database of the assembled transcriptome was established using the ViroBLAST tool [41]. The nucleotide sequence of the *PaBADH2* gene was retrieved from the transcriptome data by using TBlastNtool with the Rice BADH2 protein sequence.

### 4.5. PaBADH2 Transcript Comparative Sequence Analysis

In order to study the gene and protein sequences, the isolated nucleotide sequence of the *PaBADH2* gene was compared with previously reported *P. amaryllifolius* BADH2 (PND BADH2, gene ID: KY765936.1) and a nonfragrant rice *BADH2* (gene ID: 4345606)gene. *OsBADH2* and *PND BADH2* sequences were retrieved from the NCBI database, along with their respective amino acid sequences. Using expasy translate tools (http://www.expasy.org/) (accessed on 18 February 2019), the *PaBADH2* nucleotide sequence was translated. The sequences were aligned together with the ClustalW 1.8 program (http://www.ebi.ac.uk/clustalw/) (accessed on 18 February 2019).

### 4.6. Homology Modeling and Molecular Docking of BADH2

The amino acid sequences of PaBADH2 and nonfragrant rice (gene ID 4345606) were further used for protein structure prediction through SWISS-MODEL (https://swissmodel.expasy.org/interactive) (accessed on 18 February 2019). The homology modeling was performed using *Zea mays* AMADH (aminoaldehyde dehydrogenase) as a template. To study the enzyme–substrate interaction, BAD was taken as a substrate. The structural details of both the substrates were collected from the PubChem database. The AutoDockVina (Vina) [42] docking tool was used for ligand flexible docking simulations. The structures of the templates and the refined model of PaBADH2 were set up as the receptors for the docking protocol. The structure data file (SDF) of the ligand molecule was downloaded from PubChem (http://pubchem.ncbi.nlm.nih.gov) (accessed on 18 February 2019), and docking was performed at the acceptor and donor binding sites, as reported earlier [27]. The molecular docking was performed between OsBADH2 and PaBADH2 with BAD as a substrate.

### 4.7. Estimation of BADH Enzyme Activity

The BADH activity of 100 mg protein isolated from *P. amaryllifolius*, Basmati 370 (scented rice), and IR 64 (nonscented rice) was measured with BAD and GAB-ald as substrates following the method of Weretilnyk et al. [43].

### 4.8. Quantitative Real-Time Expression of BADH2 Gene

Total RNA was isolated from leaf tissues of Basmati 370 (scented), IR-64 (nonscented) rice varieties, and *P. amaryllifolius*. 1 μg of RNA of the selected plant species was used to carry out first-strand cDNA synthesis using RevertAidTM M-MuLV Kit (Fermentas, Waltham, Massachusetts, USA). For qPCR analysis, *PaBADH2* and *OsBADH2* gene-specific primers were designed through Primer 3.0 software (http://biotools.umassmed.edu/bioapps/primer3_www.cgi) (accessed on 18 February 2019) (Table 1). The PCR conditions for the BADH2 were optimized using gradient PCR (Eppendorf, Hamburg, Germany) for real-time expression analysis. The BADH2 expression analysis was performed in the Mastercycler ep realplex system (Eppendorf, Hamburg, Germany) using iQ SYBR Green Supermix (Bio-Rad, Hercules, CA, USA). The real-time quantitative PCR was carried out in a total volume of 25 μL containing 12.5 μlSYBR Green, 1 μL of each forward and reverse primer (10 pmol/μL), 1μL of Cdna, and 9.5 μL water. The PCR conditions were kept as follows: 95 ^0^C for 10 min one cycle; 95 °C, 30 s; 56 °C, 30 s; 72 °C, 1 min for 40 cycles were performed in triplicates and repeated twice. The diluted cDNA (1:4) was amplified with the primers based on a gene of interest, and the Ct values generated were used to prepare the standard curve. Initial copy numbers of unknown samples were calculated by using Step One Real-Time software absolute quantification model. *The elongation factor 1 (EF1)* housekeeping gene was used as an internal control to normalize the expression of the target gene, and relative expressions of *BADH2* were measured following the 2^−ΔΔCT^ method [44]. The transcript abundance was calculated as fold change/10 ng of cDNA.

### 4.9. Quantification of 2AP Using HS-SPME with GC-MS

The quantification of 2AP in *P. amaryllifolius* and aromatic rice seeds was carried out using HS-SPME with GC-MS, as previously described by Wakte et al. [8] and Hinge et al. [45], respectively. Briefly, a 4 mL screw-top vial (15 × 45 mm) with PTFE silicone septa (Chromatography Research Supplies, Louisville, KY, USA) was used to take 100 mg leaf material (powdered) of *P. amaryllifolius*. The samples were incubated for 10 min at 80 °C for equilibration. The 1 cm-long SPME fiber coated with carboxen/divinyl-benzene/poly-dimethyl-siloxane (CAR/DVB/PDMS) was used for adsorption of volatiles for 25 min. the volatile compounds were recognized with respect to the presence of specific ions, their respective ratio, and comparing the MS spectra with the reference spectra from the National Institute of Standards and Technology (NIST, ver. 2.0f, 2008) mass spectral database. A series of n-alkanes (C8 to C20) was used for calculating retention indices [46]. The 2AP concentration was measured using an external standard method with 2,4,6-trimethylpyridine (TMP) as a reference standard [47].

### 4.10. Quantification of Proline, Methylglyoxal, and GABA

GABA and proline concentration in leaf samples of *P. amaryllifolius*, Basmati 370 (scented rice), and IR 64 (nonscented rice) were estimated using the standard method described by Gay et al. [48] and Bates et al. [49], respectively. For the estimation of methylglyoxal, initial isolation was carried out following the method of Yadav et al. [21], and quantification was performed according to Wild et al. [50].

P5CS enzyme activity was assayed following the method of García-Ríos et al. [51], with minor modifications. The *P5CS* gene expression analysis was carried out in a similar way, as described for the *BADH2* gene using *P5CS* gene-specific primers.

## 5. Conclusions

In the present study, we report a full-length functional sequence of the *PaBADH2* gene from transcriptome data of *P. amaryllifolius*. Further qPCR and BADH2 enzyme activity confirmed the functionality of PaBADH2 protein. *P. amaryllifolius* is considered to accumulate 2AP to the highest levels. Similarly, the metabolites and enzymes related to 2AP biosynthesis were also in high concentration. Although there is the presence of functional BADH2 protein, 2AP synthesis is abundant, which suggests that a different molecular mechanism might be involved in 2AP biosynthesis in *P. amaryllifolius*, which still needs to be explored. This mechanism can further be employed in the breeding of rice to develop an aromatic rice variety that is resistant to yield loss under stress conditions. The high concentrations of proline may support the generation of high concentrations of GAB-ald in sufficient concentrations so that the presence of an active BADH2 converting the GAB-ald to GABA may not deplete the GAB-ald to levels that prevent reaction with the high concentrations of methylglyoxal to produce 2AP.

## Figures and Tables

**Table 1:** Primers used for *PaBADH2* real-time expression analysis.

Name of Primer	Primer Sequences
F *Pan BADH2* (Pandanus)	5′TGTTGTAAGTCAAGGACAGTATGC3′
R *Pan BADH2* (Pandanus)	5′CCGCCCACCTCCAAATAATATAG3′
F Rice *BADH2* (Rice)	5′ ATTTGTATCTACCGCCAAAAGC3′
R Rice *BADH2* (Rice)	5′CGACATCAGTAATGATTGTGGGT3′
F Pan *P5CS* (Pandanus)	5′GAGGCAGCAACAAGCTTGT3′
R Pan *P5CS* (Pandanus)	5′GTGTACAAGAAGGGTTTCCAT3′
F Rice *P5CS* (Rice)	5′GAAGTGGTAATGGTCTTCTC3′
R Rice *P5CS* (Rice)	5′AGCAAATCTGCGATCTCATC3′

## Data Availability

The data presented in this study will be openly available after one year of publication. The SRA sequence have been deposited in NCBI with the project ID: PRJNA692823.

## Supplementary Materials

The following are available online at https://www.mdpi.com/article/10.3390/ijms22136968/s1, Table S1: Primary assembly statistics of the *P. amaryllifolius* transcriptome.
