# Supplementary material for: Fragrance in Pandanus amaryllifolius Roxb. Despite the Presence of a Betaine Aldehyde Dehydrogenase 2
Source: Int J Mol Sci. 2021 Jun 28;22(13):6968. doi: 10.3390/ijms22136968 (PMC8269274; doi:10.3390/ijms22136968)
Supplement: Supplementary file 1 [file ijms-22-06968-s001.zip › ijms-1214012-supplementary.pdf]

Parameters	Sample
N75	555
N50	1010
N25	1663
Minimum transcript length	233
Maximum transcript length	9806
Average transcript length	802
total number of transcripts	34672
Total number of mapped sequence reads	27811267
GC%	46

Supplementary Table S1. Primary assembly statistics of the *P. amaryllifolius* transcriptome.
